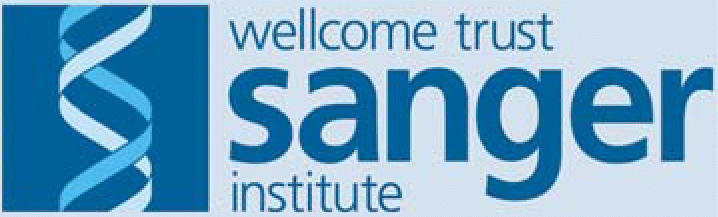# EHPnet: Wellcome Trust Sanger Institute

**Published:** 2006-03

**Authors:** Erin E. Dooley

The Cambridge, United Kingdom–based Wellcome Trust Sanger Institute (WTSI) was founded in 1993 to provide a center for research on the genomes of humans and other organisms. The institute has been a leading contributor to the Human Genome Project and now focuses much of its work on studying human DNA sequence variations and how these correlate with genetic diseases. The institute has developed a website located at **http://www.sanger.ac.uk/** to serve as a central repository for information on its varied areas of study.

The densely packed website offers a wealth of scientific resources related to its research on human genetics, model organisms, pathogens, bioinformatics, sequencing, and proteomics. Visitors also have several points of access to databases including Ensembl, COSMIC, Pfam, GeneDB, Wormbase, Vega, *MEROPS*, and DECIPHER.

Clicking on any of the topic headers in the center of the homepage leads to an assemblage of resources, project information, and laboratory homepage links related to the WTSI’s six main areas of study. Clicking on Human Genetics, for example, takes visitors to a lengthy menu of team links, each of which leads to information on what that team is working on, along with relevant references and illustrations. The Model Organisms link yields resources categorized by organism: mouse, zebrafish, *Caenorhabditis elegans*, *Schizosaccharomyces pombe*, and *Xenopus tropicalis*. The Pathogens link leads to organism-specific sequencing information for more than 100 bacteria, fungi, protozoa, helminths, vectors, and plasmids, as well as a variety of computational tools. The Bioinformatics header leads to links for downloading a number of programs, including packages for production sequencing, physical mapping, informatics analysis, and special data descriptions and specifications used at the WTSI. The Sequencing link pulls together in one area those resources specific to sequencing of various organisms. And the Proteomics link offers information on three groups at the institute that are working in this field of study.

Visitors can also navigate through a bar down the left-hand side of the homepage, which offers quick links to biological resources, the database resources mentioned above, and news releases from the institute. Biological resources listed here include the Mutagenic Insertion and Chromosome Engineering Resource and the Sanger Institute Gene Trap Resource (both of which aid in developing genetically modified animal models), the WTSI Microarray Facility (which produces arrays for a range of organisms largely for use by Sanger researchers), and Clone Ordering (which allows external scientists to order mouse and human clone cells free of charge).

Delving deeper into the site through the Site Map, visitors will find a page on Functional Genomics projects including the Human Epigenome Project (HEP), a public–private consortium of the WTSI, Epigenomics AG, and France’s Centre National de Génotypage. The goal of the HEP is to locate, identify, and catalog methylation variable positions within the human genome. The partners have conducted a pilot study of the methylation patterns within the major histocompatibility complex, a region of chromosome 6 that is associated with more diseases than any other region of the human genome. An overview of this pilot study is available within the HEP section, along with a look at the data analysis process employed by this study, information on epigenotyping, and links to the HEP partners.

## Figures and Tables

**Figure:**